# A Carbon Foam with Sodiophilic Surface for Highly Reversible, Ultra‐Long Cycle Sodium Metal Anode

**DOI:** 10.1002/advs.202003178

**Published:** 2020-12-04

**Authors:** Xue‐Yang Cui, Ya‐Jing Wang, Hua‐Deng Wu, Xiao‐Dong Lin, Shuai Tang, Pan Xu, Hong‐Gang Liao, Ming‐Sen Zheng, Quan‐Feng Dong

**Affiliations:** ^1^ Collaborative Innovation Center of Chemistry for Energy Materials (iChEM) State Key Laboratory of Physical Chemistry of Solid Surfaces Department of Chemistry College of Chemistry and Chemical Engineering Xiamen University Xiamen 361005 China; ^2^ College of Chemistry Zhengzhou University Zhengzhou 450001 China

**Keywords:** carbon materials, coulombic efficiency, long cycle life, no dendrite formation, sodium metal anodes

## Abstract

Sodium metal anodes combine low redox potential (−2.71 V versus SHE) and high theoretical capacity (1165 mAh g^−1^), becoming a promising anode material for sodium‐ion batteries. Due to the infinite volume change, unstable SEI films, and Na dendrite growth, it is arduous to achieve a long lifespan. Herein, an oxygen‐doped carbon foam (OCF) derived from starch is reported. Heteroatom doping can significantly reduce the nucleation resistance of sodium metal; combined with its rich pore structure and large specific surface area, OCF provides abundant nucleation sites to effectively guide the nucleation and subsequent growth of sodium metal, and the nature of this foam can accommodate the deposited sodium. Furthermore, a more uniform, robust, and stable SEI layer is observed on the surface of OCF electrode, so it can maintain ultra‐high reversibility and excellent integrity for a long time without dendritic growth. As a result, when the current density is 10 mA cm^−2^, the electrode can maintain stable 2000 cycles and the coulombic efficiency can reach to 99.83%. Na@OCF||Na_3_V_2_(PO_4_)_3_ full cell also has extremely high capacity retention of about 97.53% over 150 cycles. These results provide a simple but effective method for achieving the safety and commercialization of sodium metal anode.

## Graphical abstract

An O‐doped carbon foam with a sodiophilic surface is constructed and applied to the sodium metal anode. The excellent sodiophilic property, abundant porous structure, and large specific surface make the material stable during multiple plating/stripping processes, and no sodium dendrites are formed. Consequently, the sodium metal battery exhibits excellent electrochemical performance.

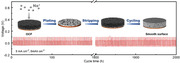

## 1 Introduction

Energy issue is a major challenge facing human society today. Therefore, the development of high‐energy density storage systems is the current market demand. Lithium‐ion batteries are currently the most widely used energy storage devices in portable electronic equipment, like smartphones and laptops, due to the superior energy density and cycle life to the lead–acid or zinc–manganese batteries. However, the insufficient lithium resources and high cost may hinder the large‐scale applications in electric vehicles.^[^ ^1^ ^]^ Sodium‐ion batteries are expected to be an ideal alternative to lithium‐ion batteries, as sodium is abundant, widely distributed, and has a suitable electric potential. Moreover, sodium and lithium are both alkali metals, and thus share many similarities in chemical properties and follow similar principles and mechanisms in terms of electrochemistry.^[^ ^1^, ^2^ ^]^

At present, general anodes for sodium batteries such as hard carbon,^[^ ^3^ ^]^ alloy compounds,^[^ ^4^ ^]^ metal oxides,^[^ ^5^ ^]^ metal sulfides,^[^ ^6^ ^]^ phosphorus,^[^ ^7^ ^]^ etc., have the capacity limited by the above existing framework, while sodium metal anodes without hosts are based on plating/stripping reactions and thereby could achieve high theoretical capacity. As the anode material of sodium batteries, sodium metal has the advantages of low redox potential (−2.71 V vs SHE) and high theoretical capacity (1165 mAh g^−1^),^[^ ^8^ ^]^ Moreover, Na metal anodes are one of the key components of high‐energy‐density Na‐based batteries, such as ZEBRA batteries,^[^ ^9^ ^]^ Na–S batteries,^[^ ^10^ ^]^ Na–O_2_ batteries.^[^ ^10^, ^11^ ^]^

However, sodium metal anode suffers the following problems: 1) the growth of sodium dendrites and the short‐circuit problems; 2) formation of unstable solid electrolyte interface (SEI); 3) infinite volume changes during the cycle.^[^ ^12^ ^]^ Regulation of the SEI components was proved to be effective to minimize the parasitic reaction between Na metal and the electrolyte, thereby improving the coulombic efficiency. Cui's group compared the combination of different electrolyte salts and solvents and found that NaPF_6_ in glymes can form a uniform, thin SEI containing Na_2_O and NaF. It could enable non‐dendritic plating–stripping cycles of Na metal anodes at room temperature.^[^ ^13^ ^]^ In addition, the structure and surface physicochemical properties of the current collector are crucial to reversibility of Na plating and stripping process. The construction of nanostructured current collectors have been implemented with large surface area, including 3D copper nanowire current collectors,^[^ ^14^ ^]^ porous Al foils,^[^ ^15^ ^]^ porous 3D Ni,^[^ ^16^ ^]^ and chemically engineered porous copper (Cu) matrix.^[^ ^17^ ^]^ Besides, the deposition host engineering can well limit the sodium metal to maintain the integrity of the electrode.^[^ ^18^ ^]^ So far, various 3D structural materials have been used for sodium plating hosts. Hu's group applied carbonized wood as a host to form a processable and moldable composite Na metal anode with stable cycling performance over 500 h at 1.0 mA cm^−2^.^[^ ^19^ ^]^ Researchers named the Au–Na alloy “sodiophilic” material, which possessed good affinity with Na. They could minimize the nucleation barrier and render uniform nucleation of Na and further uniform plating.^[^ ^20^ ^]^ Lately, some 3D materials with sodiophilic surfaces were reported, such as N‐,^[^ ^21^ ^]^ S‐,^[^ ^22^ ^]^ and O^[^ ^23^ ^]^‐doped carbon materials, which could make more use of the surface area and the physical room of the 3D host to accommodate plated Na to mitigate the volume expansion. Despite all aspects of optimization, the research of sodium metal anode is still focused on its infancy compared with lithium metal anode, considering its low plating capacity, current density, and short lifespan. In particular, under high current density and high deposition capacity, long‐cycle‐life without dendrite growth is maintained.^[^ ^24^ ^]^ This is because the high overpotential during metal plating with high current density reduces the critical radius of nucleation, a large number of nuclei on the surface of the current collector will cause the grain size of the plated metal sodium to decrease, and then a rough and uneven surface will be formed, which will generate sodium dendrites and even dead sodium, affecting the electrochemical performance of the material.^[^ ^25^ ^]^ Therefore, it is meaningful to develop an extremely stable electrode with high surface area and good sodiophilic property for highly reversible sodium metal anode.

**Figure 1:**
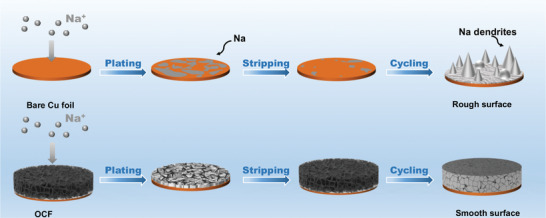
Schematic illustration of the metallic Na stripping/plating on the bare Cu foil and OCF electrodes.

Here, we introduce a sodium metal anode based on an O‐doped carbon foam (OCF) with an open 3D cross‐linked netlike porous structure and sodiophilic surface, which was synthesized by a template method using the water‐soluble starch as raw material. This material has the advantages of common raw materials, convenient synthesis, and low cost. More importantly, OCF has a large specific surface area of 1088.2 m^2^ g^−1^ with abundant reticular micropores and mesoporous structures, which has good integrity, reversibility, and characteristics of accommodating deposited metal Na, allowing repeated plating/stripping processes. Due to the benefits of raw materials and synthesis methods, O heteroatom is introduced to form a sodiophilic nucleation layer, so that the electrode showed a smaller nucleation overpotential and the following metal growth was uniform and compact with much more nucleation sites. The sodiophilic surface, rich pore structure, and large specific surface area of OCF provide sufficient space for deposited metal Na. In addition, a more uniform, stable, and robust SEI is the guarantee of long cycle stability. Therefore, the OCF electrode exhibits excellent reversibility and stability, and can still form a dense and smooth Na metal layer after multiple stripping and plating processes without the formation of dendrites. In contrast, due to the weak sodiophilic property of copper foil and uneven surface condition, the unconstrained uneven deposition and growth of metallic Na gradually lead to the formation of Na dendrites (**Figure** **1**). High current density usually leads to non‐uniform Na^+^ flux, which is prone to facilitate the uneven deposition, even dendrite growth. In view of the above advantages of the OCF, electrode can exhibit high coulombic efficiency of 99.90% for 2000 h at 5 mA cm^−2^ with a fixed areal capacity of 5 mAh cm^−2^. Even when the current density is as high as 10 mA cm^−2^, the coulombic efficiency of 99.83% can still be maintained. The full cells assembled by Na@OCF anode and Na_3_V_2_(PO_4_)_3_ cathode also exhibits outstanding cycle stability.

## 2 Results and Discussion

### 2.1 Structural and Morphological Characterizations

**Figure 2:**
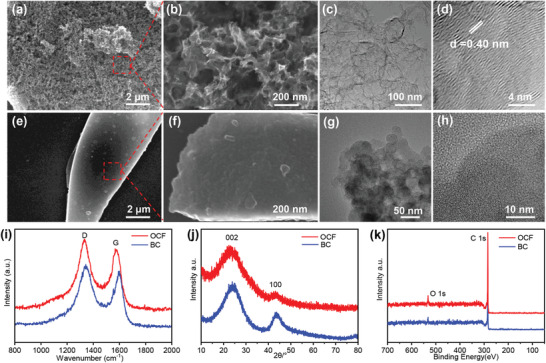
SEM images of a,b) OCF and e,f) BC, and HRTEM images of c,d) OCF and g,h) BC. i) Raman spectra; j) XRD patterns; k) the full XPS spectra of the OCF and BC.

The morphologies of the OCF and BC (bulk carbon material without template) were observed by scanning electron microscope (SEM) as shown in **Figure** **2**a–h. After adding the template, the OCF obviously shows a 3D cross‐linked network porous structure, while the SEM images of BC reveals no morphological features with the large bulk stack and rough surface. High‐resolution transmission electron microscopy (HRTEM) images also show that the OCF has the mesoporous mesh structure and exhibits disordered amorphous peculiarity. There are several graphitized local structures in this material and the measured lattice spacing is 0.40 nm. This abundant and open porous structure and interconnected channels promote electrolyte penetration into the material to accelerate the kinetics of ion diffusion.^[^ ^26^ ^]^ Moreover, they also provide abundant Na anchoring sites. However, for BC, there is no template to inhibit the development of ordered carbon structure, which indicates the higher graphitization feature.^[^ ^26^, ^27^ ^]^

To investigate further the crystalline structural information of samples, Raman spectroscopy and X‐ray diffraction (XRD) analysis were utilized. The Raman spectrum shows that the D and G bands are located at 1332 and 1575 cm^−1^, respectively, which are related to the defects or disorders of the hexagonal graphite layer and in‐plane stretching vibration of C atom sp^2^ hybridization.^[^ ^27^ ^]^ Moreover, we generally use the intensity ratio of D peak and G peak to measure the edge and topological defect degree of materials.^[^ ^28^ ^]^ The results show the *I*_D_/*I*_G_ of OCF is 1.14, which is greater than 1.03 of BC, indicating a higher disorder degree of OCF (Figure 2i). Furthermore, the XRD pattern of the OCF and BC show conspicuous large drums at 24° and 43° instead of sharp peaks, which correspond to (002) and (100) plane in amorphous carbon structure, respectively (Figure 2j). According to the location of the (002) peaks, the interlayer spacing (*d*‐value) can be calculated. Due to the (002) peak of OCF shifts to a lower angle, the layer spacing *d*_002_ of the OCF is calculated to be 3.800 nm, which is larger than the BC layer spacing *d*_002_ of 3.588 nm. The aforementioned results of XRD and Raman spectroscopy are consistent with HRTEM characterization, indicating that the nano‐CaCO_3_ template will affect the annealing process and form a higher degree of disordered carbon.^[^ ^27^ ^]^

The specific surface area and pore structure of OCF are shown by the nitrogen adsorption–desorption isotherm curves, as shown in Figure S1a,c, Supporting Information. It can be seen that the OCF adsorption–desorption isotherm belongs to the pseudo‐type II isotherm and the H3 hysteresis loop appears.^[^ ^29^ ^]^ The specific surface area of the OCF is measured to be 1088.2 m^2^ g^−1^, and the specific surface area of the BC is 224.6 m^2^ g^−1^ (Figure 2m). The Barrett–Joyner–Halenda (BJH) pore size distribution shows that the OCF is mainly micropores and a few mesopores locating in 0.5–2 nm and 2–100 nm. The pore volume of the OCF is calculated to be 1.355 cm^3^ g^−1^, and the BC pore volume is 0.116 cm^3^ g^−1^. Common BC has only some micropores, mainly because the addition of the template promotes the formation of mesopores and macropores. In addition, it has been confirmed by X‐ray photoelectron spectroscopy (XPS) that the OCF material contains a certain amount of O heteroatom doping (Figure 2k), and the C/O ratio is 12.5 and 17.2 in OCF and BC, respectively. In the C 1s spectra, C—C, C—OH, C—O—C, and COOH bondings were observed (Figure S2, Supporting Information). O atoms with stronger electronegativity increase the polarity of OCF materials, so that they exhibit better sodiophilic property^[^ ^30^ ^]^ and are beneficial to exhibit better electrochemical performance.

### 2.2 Electrochemical Performances of the OCF Electrode

**Figure 3:**
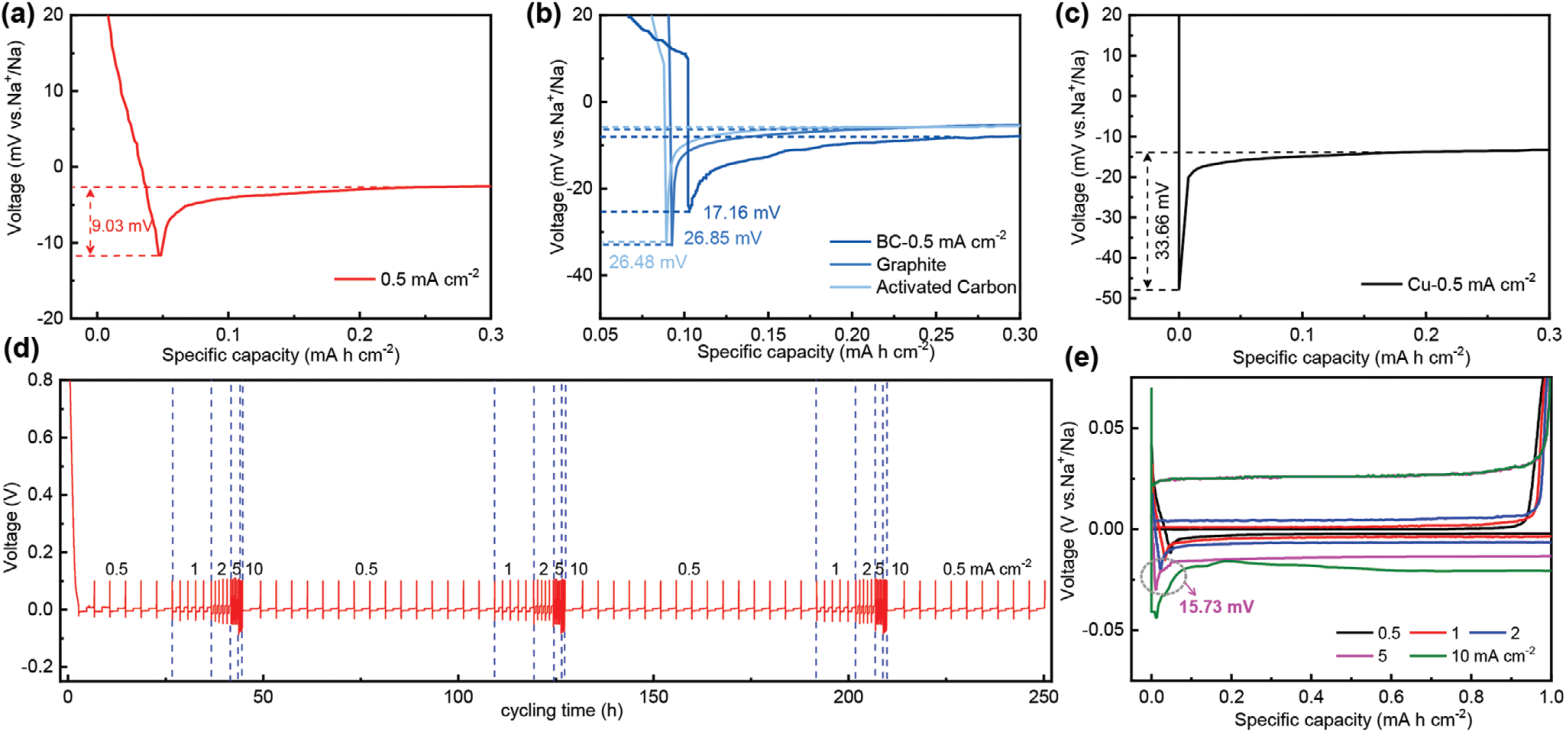
a) Galvanostatic sodium metal plating profiles at a current density of 0.5 mA cm^−2^ on OCF electrode; b) BC electrode and graphite electrode; c) Cu electrode. d) Galvanostatic sodium metal plating/stripping profiles at different current densities from 0.5 to 10 mA cm^−2^; e) rate performance of the OCF at various current densities of 0.5–10 mA cm^−2^.

The electrochemical performance was tested by galvanostatic plating and stripping of Na metal measurement. **Figure** **3**a–c showed the voltage–capacity profiles. We can see a sudden drop in voltage during the Na metal plating process, followed by a flat Na plating voltage platform. The Na metal nucleation overpotential refers to the voltage difference from the lowest point of the voltage dip to the voltage platform; this value can reflect the nucleation barrier of the material.^[^ ^31^ ^]^ The doping of heteroatoms O effectively reduces the nucleation barrier and promotes the adsorption and uniform distribution of Na^+^ on the electrode surface.^[^ ^32^ ^]^ Especially, carboxyl oxygen is the best strategy for monatomic doping, because doping sites with larger local dipoles form larger ion–dipole interaction toward Na ions, thus showing larger binding energy and smaller nucleation barrier, and there is obvious charge transfer from Na atom to carboxyl oxygen.^[^ ^33^ ^]^ Therefore, OCF (≈9.03 mV) and BC (≈17.16 mV) electrode exhibited lower nucleation overpotential than graphite (≈26.85 mV), activated carbon (≈25.48 mV) electrode, and Cu foil (≈33.66 mV) when the current density was 0.5 mA cm^−2^. In addition, due to the large specific surface area, the local current density is greatly reduced, and the nucleation overpotential is also significantly reduced, which is favorable for uniform deposition of Na and reduces the initial Na metal nucleation size to inhibit the formation of Na dendrites. This is also the reason why the nucleation overpotential of OCF is the lowest. Besides, we observed the rate voltage profiles on OCF electrode of Na metal plating/stripping at different current densities of 0.5–10 mA cm^−2^ in Figure 3d,e. It could be seen that the overpotential gradually increases with the increase of current density. The OCF electrode exhibited relatively lower nucleation overpotentials of 9.03, 11.4, 12.7, and 15.7 mV at 0.5, 1, 2, and 5 mA cm^−2^, respectively. Even at the current density of 5 mA cm^−2^, the nucleation overpotential remained within ≈15.73 mV, which was much lower than the nucleation overpotential of the BC electrodes at the same current densities (Figure S3, Supporting Information). Ordinarily, metal nucleation can be described by the following first‐order linear process: d*N*/dt + *AN* = *AN*_0_, *N*(0) = 0; *N*_0_, *N*, and *A* represent the number density of active sites, the density, and nucleation rate of nucleated particles, respectively. Low overpotential range forms a kinetically controlled metal growth regimes (*k*_G_/D*N*_0_^1/2^ << 1) which allows metal to grow uniformly on the electrode surface, when the overpotential increases to form a diffusion control (*k*_G_/DN_0_^1/2^ >> 1); it is usually accompanied by the growth of dendrites (D: diffusion coefficient; *k*_G_: charge transfer kinetic constant; *N*_0_: number density of the active sites). Therefore, increasing the active sites can effectively affect the size, morphology, and growth mechanism of the nucleation particles.^[^ ^23^, ^26^ ^]^ Therefore, open 3D OCF with quantities of nucleation sites and a large specific surface area can effectively reduce the nucleation barrier, achieve uniform deposition of Na metal, and provide excellent high‐rate performance.

**Figure 4:**
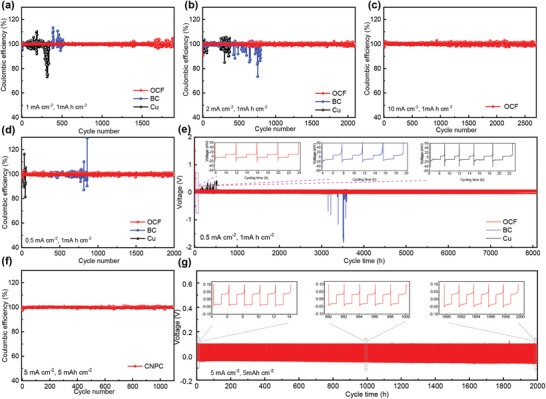
Coulombic efficiency of different electrode under different current densities with capacity limitations of 1.0 mAh cm^−2^: a) 1 mA cm^−2^, b) 2 mA cm^−2^, c) 10 mA cm^−2^, d) 0.5 mA cm^−2^. e) Cycling performances of OCF, BC, and Cu foil at a current density of 0.5 mA cm^−2^ with a capacity of 1 mAh cm^−2^. f) Coulombic efficiency of OCF electrode and g) cycling performances at a current density of 5 mA cm^−2^ with a capacity of 5 mAh cm^−2^.

In general, we use the ratio of Na stripping capacity to Na plating capacity, which is coulombic efficiency (CE), to evaluate the reversibility of the Na metal plating/stripping process. The average coulombic efficiency at different current densities of 0.5, 1, and 2 mA cm^−2^ with a capacity limitation of 1.0 mAh cm^−2^ on OCF electrodes was as high as 99.90%, 99.89%, and 99.94%, respectively (**Figure** **4**a,b,d). In particular, when the current density was up to 10 mA cm^−2^, the coulombic efficiency still was maintained at 99.83% over 2500 cycles (Figure 4c). The high CE of OCF electrode is vitally important to high capacity retention of full cells.^[^ ^34^ ^]^ However, the coulombic efficiency of Na metal on the BC and bare Cu foil showed significant fluctuations after a few hundred cycles, resulting in a gradual reduction of reversible capacity, which was due to an uneven Na plating/stripping on the electrode that tends to short‐circuit more easily. This also reflected that the interface of BC and Cu foil deteriorated continuously and even generated dead sodium during long cycle. Additionally, we observed the cycle performance between three different electrodes of OCF, BC, and Cu foil at a current density of 0.5 mA cm^−2^ with a plating capacity of 1.0 mAh cm^−2^ as shown in Figure 4e. By comparing the long cycle curve and voltage curve at the same time, it was found that both Cu foil and BC showed large irregular voltage fluctuations after a period of time, which indicated that the formation of Na dendrites even caused short circuits, while the OCF electrode had a lower nucleation overpotential, a smoother voltage curve, and a longer cycle life. More notably, when the current density was as high as 5.0 mA cm^−2^ with a capacity of 5.0 mAh cm^−2^, the OCF electrode still maintained ultrahigh cycle stability as shown in Figure 4g. The OCF electrode at 5 mAh cm^−2^ has a gravimetric capacity of 1055 mAh g^−2^_Na@OCF_, which is very close to the theoretical capacity of metal Na anode of 1167 mAh g^−2^_Na_. Additionally, the detailed voltage profiles of different cycles were embedded in this figure; we observed that the plating/stripping process of metal Na exhibited a flat voltage platform and the overpotential had no obvious change over 2000 h, and the coulombic efficiency remained as high as 99.90% (Figure 4f). Through the above results, we realize that OCF electrode can solve the problem of long cycle difficulty of Na metal cathode under high surface capacity and high current density. Its electrochemical performance is superior to other reported work, whether in high current density, lifespan, or coulombic efficiency (Table S2, Supporting Information). Generally, under low temperature conditions, sodium metal anodes tend to intensify dendrite growth due to the slow migration of sodium ions.^[^ ^35^ ^]^ However, the rich and dense open cross‐networked porous structure can promote electrochemical kinetics, improve the ion self‐concentrating kinetics, and make ion distribution uniform,^[^ ^36^ ^]^ which makes it possible to maintain dendrite‐free growth and high cycle stability at a current density of 1 mA cm^−2^ with a low temperature of −5 °C on OCF electrode (as shown in Figure S4, Supporting Information).

### 2.3 Uniform Na Stripping/Plating Behavior and Morphology Evolution of OCF Electrode

**Figure 5:**
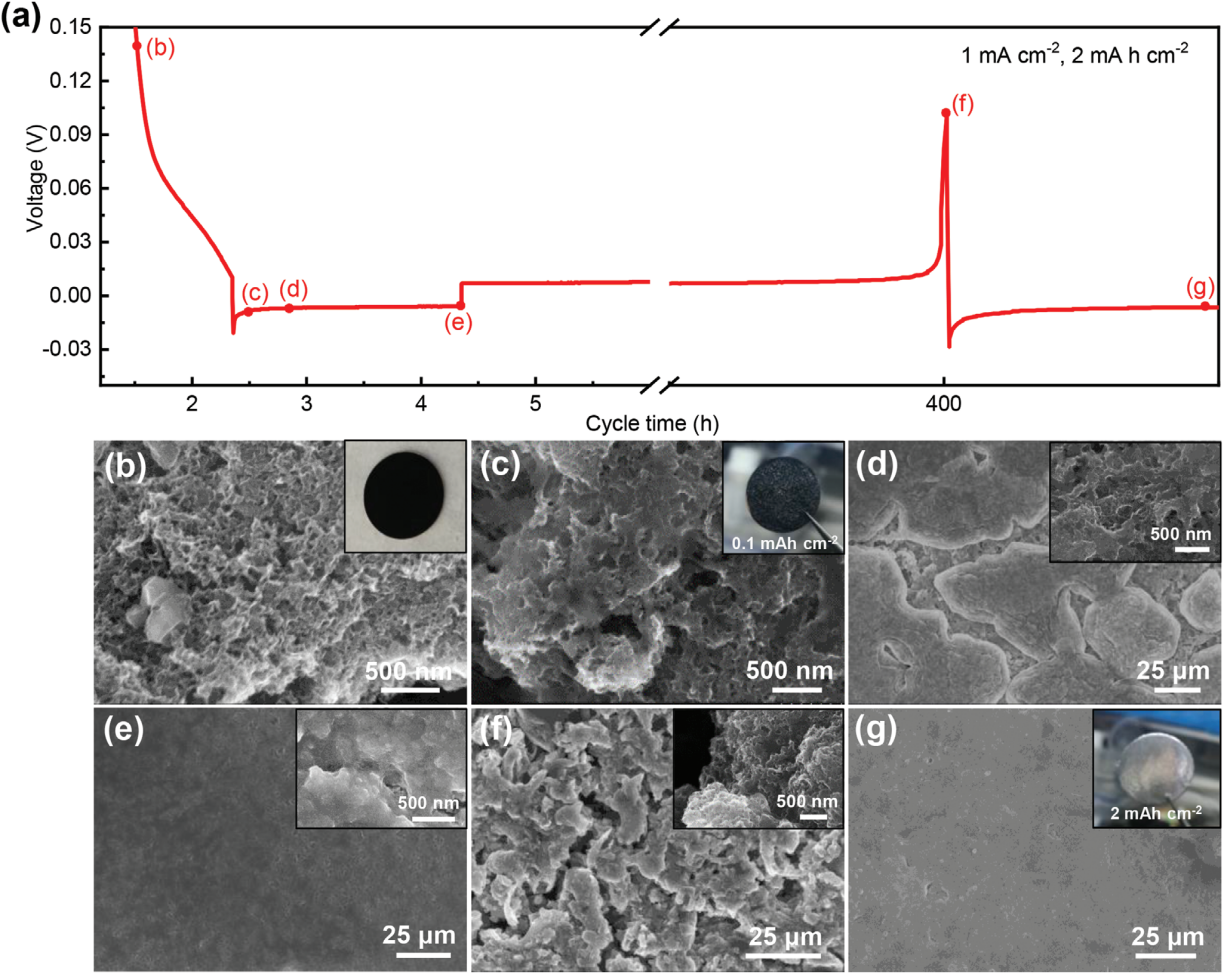
a) Galvanostatic plating/stripping of OCF electrodes at current densities of 1 mA cm^−2^ with a capacity of 2 mAh cm^−2^. Top‐view SEM images of the OCF electrode before plating/stripping (b) and Na metal on the OCF electrode with different plating capacities c) 0.1 mAh cm^−2^, d) 0.5 mAh cm^−2^, e) 2.0 mAh cm^−2^; f) Na metal stripped after 100 cycles; g) Na metal plated 2.0 mAh cm^−2^ after 100 cycles.

We further observed in detail the plating/stripping behavior of metallic Na with a constant current of 1.0 mA cm^−2^ on the OCF electrode. Before the deposition of sodium metal, the electrode completely maintained its open 3D cross‐linked network porous structure; when the plating capacity was as small as 0.1 mAh cm^−2^, it could be seen that metal Na was uniformly dispersed on the surface of OCF from the optical picture, and the open network structure of OCF can still be observed through the top‐view SEM image (**Figure** **5**b,c). With the increase of the plating capacity, metal Na can fill the pore structure and then grow in the horizontal direction, and the surface of the electrode gradually covers the Na metal island. When the plating capacity reached 2 mAh cm^−2^ (Figure 5d,e), Na metal would smoothly cover the entire electrode surface. Thanks to the sodiophilic property and large specific surface area of OCF, the uniform distribution of Na^+^ flux on OCF electrodes is promoted and metallic Na is easily and uniformly deposited on the surface of OCF electrodes.^[^ ^37^ ^]^ Moreover, we also performed a surface wettability experiment of liquid metal Na on the surface of OCF electrode. As shown in Figure S5, Supporting Information, we saw that metal Na can be completely dispersed on the surface of OCF, which proved the excellent sodiophilic property of OCF material. After ≈400 h, a uniform and smooth Na metal layer could still be observed on the surface of the OCF electrode without the formation of Na dendrites (Figure 5g). Therefore, not only can the OCF affect the nucleation process, but also effectively guide the subsequent growth process, thus regulating the growth of dendrites. As for the bare Cu electrode (Figure S6, Supporting Information), from the optical picture, when the plating capacity was 0.1 mAh cm^−2^, the uneven distribution of the metal Na could be clearly seen. Through the top‐view SEM images, we clearly observed the microscopic morphology of the Na plating process on the Cu foil. Unlike the OCF electrode, when the plating capacity reached 2 mAh cm^−2^, Na metal covered the entire Cu electrode, but with visible micron‐sized pores and cracks, demonstrating the uneven deposition of Na metal on the Cu electrode. The surface morphology of Na metal formed on the electrode is closely related to the electrochemical performance.^[^ ^38^ ^]^ This further illustrated the advantages of heteroatom doping, suitable porous structure, and large specific surface area on OCF electrode, which makes it effortless to form uniform Na^+^ flux and guide sodium metal nucleation and uniform growth. Therefore, the OCF electrode exhibits excellent electrochemical stability and rate performance.

**Figure 6:**
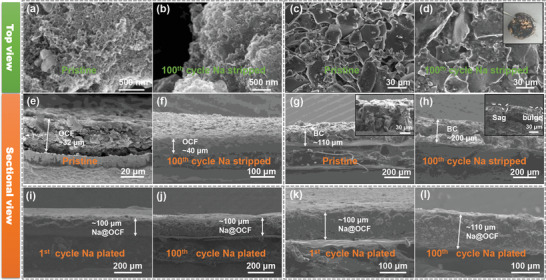
Top view SEM images of a) OCF electrode, b) OCF electrode after 100 cycles, c) BC electrode, and d) BC electrode after 100 cycles. Sectional view SEM images of e) OCF electrode, f) OCF electrode after 100 cycles, g) BC electrode, and h) BC electrode after 100 cycles OCF electrode with a current density of 1 mA cm^−2^ and a plating capacity of 2 mAh cm^−2^. i,j) Sectional view SEM images of OCF electrode after different cycles with a current density of 1 mA cm^−2^ and a plating capacity of 2 mAh cm^−2^ (1100 cycles). k,l) Sectional view SEM images of OCF electrode after different cycles with a current density of 5 mA cm^−2^ and a plating capacity of 5 mAh cm^−2^ (1100 cycles).

Then we observed the plating/stripping behavior of metal Na on different electrodes during cycling to understand the mechanism of maintaining long cycle or cyclic attenuation. Previous studies have shown that the initial Li nucleation process is crucial in Li metal, which can lead to the formation of uniform Li metal layer and provide some guidance for Na metal research.^[^ ^39^ ^]^ Electrode materials with high specific surface area and good sodiophilic property can provide abundant nucleation sites, which greatly reduces the local current density and is beneficial for regulating the deposition of metallic sodium.^[^ ^40^ ^]^ We traced the growth and deposition of Na metal on different electrodes including OCF, BC, and bare Cu foil by ex situ SEM. As shown in **Figure** **6**a–e, the pristine OCF electrode was about 30 µm and had an obviously cross‐linked netlike porous structure. These abundant micropores and mesoporous structures can provide surplus and vast space to accommodate metal Na. Then, we observed the surface and cross‐section evolution when the plating capacity was 2 mAh cm^−2^. On the OCF electrode, Na metal was uniformly deposited in the three‐dimensionally connected network porous structure. The sectional view showed that the thickness of the Na@OCF layer was about 100 µm (Figure 6i); the surface of the Na@OCF layer was very flat with no formation of dendrites, and through energy dispersive spectroscopy mappings, we found that the C and Na elements were evenly distributed in the cross‐section of the OCF electrode (Figure S7, Supporting Information). This further illustrated that metal Na can uniformly be deposited on the OCF electrode. After metal Na was stripped in the 100th cycle, the OCF still retained its original connected network porous structure as shown in Figure 6b,f, and the thickness of OCF electrode layer increased only slightly compared with the initial one. This indicates that the OCF electrode has certain elasticity and can still return to the primitive state after multiple plating/stripping processes, but when the Na metal was plated on bare Cu electrodes, the uneven electric field was caused due to the unevenness and roughness of the surface of the Cu foil. Figure S8, Supporting Information, shows cross‐section of Cu electrode. It could be seen that when the plating capacity was 2 mAh cm^−2^, the Na layer had a thickness of about 53 µm, and the surface of Na layer was rough and uneven; especially, many burrs were observed on the electrode surface, which was in sharp contrast to the Na layer on OCF electrode. Furthermore, in order to prove the importance of the cross‐linked netlike micropore and mesoporous structures, BC without template was used as a comparison for the plating/stripping of metal Na. We found that the plating/stripping process of metal Na on BC electrode also showed irreversible volume changes and defects as shown in Figure 6d,h and Figure S9, Supporting Information. After metal Na stripping, there were a large number of depressions and protrusions on the surface of the BC electrode, and the thickness of BC layer increased from ≈110 to ≈200 µm. It could be seen from the optical picture that there was still residual Na on the surface after metal Na stripping, which was also consistent with the low coulombic efficiency of the BC electrode mentioned above. It follows that the suitable structure can make the plating/stripping process of metal Na controllable.

It was well known that infinite volume changes and dendrite growth were the main causes of failure of Na‐metal batteries. The OCF electrode after 100 cycles with a plating capacity of 2 mAh cm^−2^, as shown in Figure 6b, could still maintain the integrity of the porous structure of the 3D cross‐linked netlike structures without structural collapse and cracks after multiple plating/stripping processes. By comparing the cross‐section of Na metal plating process at the first cycle and the 100th cycle (Figure 6i,j), we found that the Na metal layer maintained a similar thickness of about 100 µm and had no dendrite growth. Interestingly, when the current density was 5 mA cm^−2^ and the plating capacity was 5 mAh cm^−2^, the thickness of the Na layer after the first cycle of Na metal plating was also about 100 µm and maintained the thickness of the first cycle after 100 cycles; the surface was smooth and flat without the formation of Na dendrites (Figure 6k,l). However, for the Cu electrode, when the current density was 5 mA cm^−2^ and the plating capacity 5 mAh cm^−2^, we could observe obvious rough and uneven electrode surface and protruding fluffy sodium dendrites after 50 cycles (Figure S10, Supporting Information). These all show that the OCF electrode has special reversibility and integrity, which makes the OCF electrode able to maintain very long cycle stability as shown in Figure 5. Additionally, through the comparison of the thickness of Na layers with different plating capacities at the same current density (Figure S11, Supporting Information), it was found that when the plating capacity was 2, 3, and 5 mAh cm^−2^, the thickness of the Na layer was approximately within the range of 100 µm, which indicated the OCF structure had a highly elastic structure that can accommodate enough deposited Na metal. Elaborately, we explored the morphological evolution and electrochemical behavior of the Na metal layer under different OCF layer thicknesses and revealed the role of the OCF layer in accommodating deposited metal Na and mitigating volume changes (Figure S12, Supporting Information).

### 2.4 Observation of SEI Layer on OCF Electrode Surface

In order to further clarify the mechanism of the excellent electrochemical performance of OCF electrodes, we observed the composition and cyclic change of SEI on the electrode surface by ex situ XPS. The detailed XPS spectra of C, O, Na, are shown in Figures S13–S15, Supporting Information. Through analysis and comparison, SEI layers of the OCF, BC, and Cu electrode are similar in composition, and their main components include NaCO_3_R, NaF, and Na_2_O. In the O1s spectra, the peak of ≈529.5 eV was observed to correspond to the Na_2_O component, and the fast Na^+^ diffusion conductivity of NaF helps to form a more uniform and stable SEI layer. Based on XPS sputtering depth analysis, the characteristics of organic components on the outer surface of SEI layer, while the inner layer shows more signals of inorganic components, are similar to the SEI layer components in other ether electrolytes.^[^ ^12^, ^23^, ^41^ ^]^ The thickness of the SEI layer is about 8 nm, and it is thinner and uniform on the OCF electrode, but on the BC electrode, we can observe that the atomic ratio at different depths changes sharply, which means that the SEI layer on the BC electrode is not uniform and compact (Figure S16, Supporting Information). This makes Na metal more exposed and produces more organic by‐products with electrolyte, and greatly reduces the coulombic efficiency. By comparing the XPS spectra of SEI layer of different plating/stripping cycles, we found that the changes in the content and binding mode of each atom could be almost ignored. This stable and robust SEI layer guaranteed long cycle and high efficiency of electrodes. However, after many plating/stripping processes for BC electrodes and Cu electrodes, XPS results clearly showed obvious differences in the spectra peak. Among them, the huge change of C1s spectrum indicates that repeated plating/stripping process destroyed the already unstable SEI layer, further produced side reactions that consumed metal sodium and electrolyte, resulting in lower coulombic efficiency. Uneven SEI was more likely to form sodium dendrites or even “dead sodium.” We conducted electrochemical impedance spectroscopy measurement to further observe the changes of the electrolyte and electrode interface during the plating/stripping of metal Na (Figure S17, Supporting Information). The fitting results of equivalent circuit (R_s_(R_SEI_CPE_1_)(R_ct_CPE_2_)Z_w_) are shown in Table S1, Supporting Information. It could be found that the repeated plating/stripping process increased the interface impedance of BC electrode and Cu electrode, which was mainly due to the increase of SEI and the accumulation of dead sodium on the electrode surface. However, the stable SEI layer on the surface of OCF and excellent interfacial reaction kinetics make OCF electrode still show stable and low charge transfer resistance after 200 cycles. These results are consistent with XPS, ex situ SEM, and electrochemical performance.

### 2.5 Symmetrical Cell and Full Cell Performance of the Na@OCF Electrode

**Figure 7:**
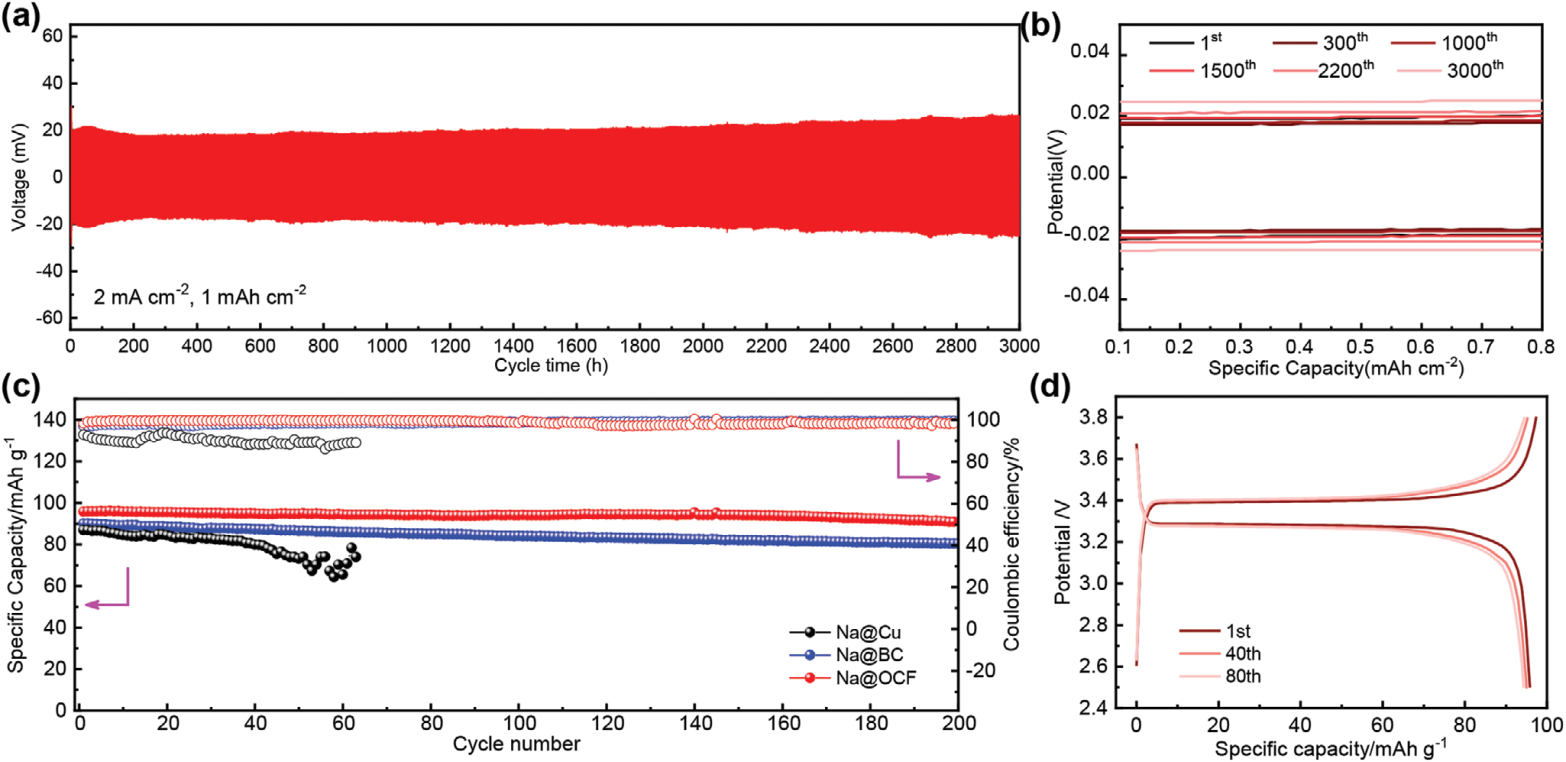
a) Galvanostatic plating/stripping of the symmetric cells of OCF electrodes at current densities of 2 mA cm^−2^ with a capacity of 1 mAh cm^−2^. b) Overpotential at different cycles. c) The cycling performances of Na|| Na_3_V_2_(PO_4_)_3_ full cells; the anode are Na@Cu, Na@BC, and Na@OCF. d) The voltage profiles of Na@OCF ||Na_3_V_2_ (PO_4_)_3_ full cells.

Subsequently, we have assembled symmetrical battery to research the long‐cycle stability of OCF electrode as shown in **Figure** **7**a,b. The OCF electrode first pre‐stored a capacity of 2.0 mAh cm^−2^, then a plating/stripping capacity of 1.0 mAh cm^−2^ with 2.0 mA cm^−2^. We observed that the OCF electrode showed cyclic stability without obvious fluctuations, and the overpotential changed very little as the cycle progresses, indicating the stability of the internal resistance. At the beginning, the OCF electrode overpotential was about 20 mV, and the overpotential was still only 25 mV after maintaining an ultra‐stable cycle of 3000 h, affirming its high stability during prolonged cycling.

The high coulombic efficiency of the OCF electrode and the stability at high plating capacity provide the possibility of achieving high‐energy‐density sodium metal batteries. In order to test the practical application performance of OCF electrode in sodium metal batteries, we used Na@OCF as anode and Na_3_V_2_(PO_4_)_3_ as cathode to assemble full cells, the OCF anode first plated with 1 mAh cm^−2^ of metallic Na. As shown in Figure 7c,d and Figure S18, Supporting Information, the voltage range was 2.5–3.8 V, and the current density was 1C (1C = 118 mA g^−1^). The capacity retention of OCF electrode was 97.53% and BC electrode was 90.99% over 150 cycles. In contrast, Na@Cu full cells showed great instability. By comparing the charge–discharge voltage curves, the Na@OCF/Na_3_V_2_(PO_4_)_3_ battery showed a smaller voltage hysteresis. This indicated the possibility of practical application of OCF electrode.

## 3 Conclusions

In summary, we have prepared an open 3D O‐doped cross‐linked netlike porous carbon material with high specific surface area and suitable porous structure by the template method, which has wide range of sources of raw materials, simple synthesis, and low cost. The results show that OCF can not only effectively guide Na metal nucleation and growth, but also has the ability to accommodate the deposited metal Na. The structural integrity and reversibility under high current density is a guarantee of good cycle stability of the electrode. As a result, the overpotential only increased to 15.73 mV at a high current density of 5 mA cm^−2^. Moreover, when the current density is 0.5, 1, 2, and 10 mA cm^−2^, the coulombic efficiency can reach 99.78%, 99.89%, 99.94%, and 99.83%, respectively. This electrode can maintain a long cycle life of 2000 h and maintain a coulombic efficiency of up to 99.90% when the current density is 5 mA cm^−2^ with the plating capacity at 5 mAh cm^−2^. In addition, through the analysis of surface chemistry on electrode by ex situ XPS, it is found that the surface of the OCF electrode has an extremely thinner, uniform, robust, and stable SEI layer. Therefore, the full cell with Na@OCF as anode exhibits extremely high cycle stability. This facile and low‐cost, high‐performance sodium metal battery material provides new opportunities for realizing the high current, high surface capacity, and long cycle life of sodium metal anodes.

## 4 Experimental Section

##### Synthesis of OCF and BC

Referring to the synthetic method of the previous work, 8 g of soluble starch was first dissolved in 50 mL of a mixed solution of ethanol and water (V:V = 1:1), stirred at room temperature, and then appropriate amount of modified nano calcium carbonate added as a template to continue stirring. The obtained homogeneous mixture was dried in an oil bath at 90 °C, then transferred to a tube furnace, and calcined at 1200 °C for 3 h under an argon atmosphere at a heating rate of 5 °C min^−1^. The product was reacted with 1 m hydrochloric acid to remove the remaining template, then washed several times with distilled water and absolute ethanol, and dried in an oven at 80 °C for 12 h to obtain OCF material. The synthesis procedure of BC was similar to OCF, except that the step of adding calcium carbonate template was removed.

##### Electrochemical Characterization

The performance of OCF in sodium metal batteries was characterized by assembling the 2032‐type cell in an argon‐filled glove box. The active material and CMC (carboxy methyl cellulose sodium) were prepared into a uniform slurry (m:m = 9:1), and then coated on a clean rough copper foil. After drying in a vacuum oven at 60 °C overnight, it was used as a working electrode. Fresh sodium foil with a thickness of 1 mm was used as counter electrode and reference electrode. The electrolyte composition was 0.01 m NaTFSI + 1.0 m CF_3_NaO_3_S in diglyme. The stripping cut‐off potential was set to 0.1 V. Galvanostatic plating and stripping of Na metal measurement was tested by NEWARE BTS‐5 V 5/20 mA type battery charger (Shenzhen NEWARE Co. LTD, China) and LANHE CT2001A; electrochemical impedance spectroscopy measurement with impedance analyzer (IM6, Zahner Elektrik, Germany) in a frequency range from 100 kHz to 0.1 Hz with an amplitude voltage of 5 mV. Then, the Na@OCF electrode was assembled into the Na/Na symmetrical battery in a glove box to test the cyclic stability performance of the metal Na anode. Finally, the full cells consisting of Na_3_V_2_(PO_4_)_3_ cathode and the Na@OCF anode were assembled and tested in the 2.5–3.8 V voltage range. The Na_3_V_2_(PO_4_)_3_ cathode was obtained by mixing active materials, Super P and PVDF, in a mass ratio of 8:1:1, using *N*‐methyl‐2‐pyrrolidinone as a solvent to obtain uniformly dispersed slurry, and coating the slurry on Al foil. The diameter of the pole piece is 13 mm, the active mass loading was 2 mg cm^−2^. The OCF, BC, and Cu electrode were plated with 1 mA h cm^−2^ of Na and then served as the anode. Celgard 2400 was used as the separator. The electrolyte for the full cell was 1 m NaPF_6_ in diglyme because of the consideration of stability of NaPF_6_ at high voltage and 35 µL of electrolyte was used in each cell. The above experiments were conducted at room temperature.

##### Other Characterization

The morphology of the material was characterized by a field emission SEM (LEO 1530, HITACHI S‐4800) and a TECNAI HRTEM (F30). The cells were disassembled in a glovebox filled with argon gas (the contents of O_2_ and H_2_O were kept below 0.1 ppm); then the working electrode was washed several times with diglyme solvent, and dried in the glove box for the next characterization. The air‐sensitive sample was sealed in a box filled with argon gas and transferred for ex situ SEM experiment. XRD patterns were collected by a Rigaku Ultima IV powder X‐ray diffractometer with Cu K*α* radiation (*λ* = 1.54056 Å) operated at 35 kV and 15 mA. The N2 adsorption/desorption isotherms were tested at 77 K by using a Micromeritics TriStar II3020 surface Area and pore analyzer. XPS measurements were carried out using PHI QUAN‐TUM 2000. For ex situ XPS tests, a commercial transfer box was used. The argon‐filled box was transferred to the chambers and then used an operation rod transferred to the test bench so that the samples were completely protected by high‐purity argon gas.

## Conflict of Interest

The authors declare no conflict of interest.

## Supporting information

Supporting Information
