# Supplementary material for: A Carbon Foam with Sodiophilic Surface for Highly Reversible, Ultra‐Long Cycle Sodium Metal Anode
Source: Adv Sci (Weinh). 2020 Dec 4;8(2):2003178. doi: 10.1002/advs.202003178 (PMC7816717; doi:10.1002/advs.202003178)
Supplement: Supplementary file 1 — Supporting Information [file ADVS-8-2003178-s001.pdf]

© 2020 Wiley-VCH GmbH

ADVANCED SCIENCE
Open Access

Supporting Information

for *Adv. Sci.,* DOI: 10.1002/advs.202003178

A carbon foam with sodiophilic surface for highly reversible, ultra-long cycle sodium metal anode

Xue-Yang Cui,‡Ya-Jing Wang,‡ Hua-Deng Wu, Xiao-Dong Lin, Shuai Tang, Pan Xu, Hong-Gang Liao, Ming-Sen Zheng, and Quan-Feng Dong**

Copyright WILEY-VCH Verlag GmbH & Co. KGaA, 69469 Weinheim, Germany, 2016.

Supporting Information

A carbon foam with sodiophilic surface for highly reversible, ultra-long cycle sodium metal anode

Xue-Yang Cui,‡Ya-Jing Wang,‡ Hua-Deng Wu, Xiao-Dong Lin, Shuai Tang, Pan Xu, Hong-Gang Liao, Ming-Sen Zheng, and Quan-Feng Dong**

‡These authors contributed equally to this work.

*Corresponding author e-mail: mszheng@xmu.edu.cn; qfdong@xmu.edu.cn

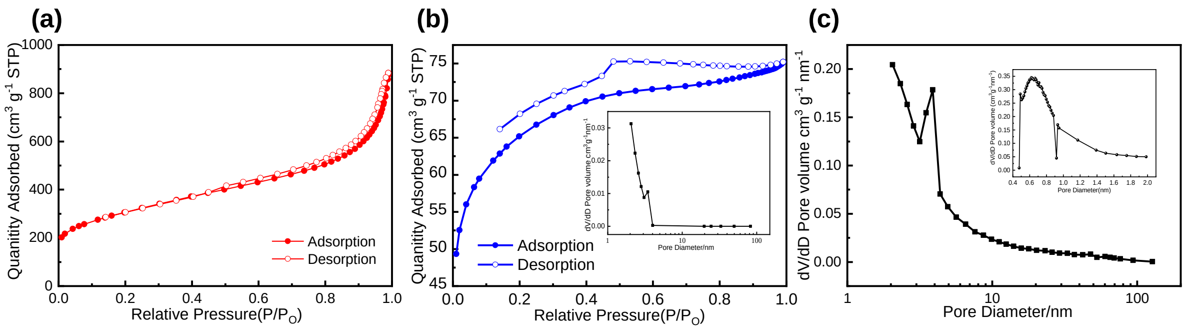

Figure S1. (a) Nitrogen adsorption and desorption isotherm curves of OCF, (b) nitrogen adsorption and desorption isotherm curves of BC and the inset shows pore size distribution, (c) pore size distribution data of OCF.

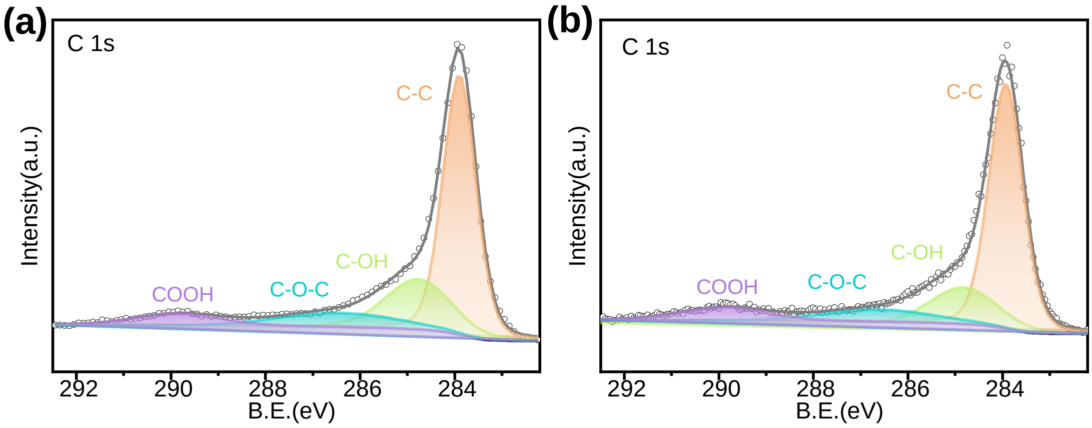

Figure S2. The C 1s spectra of pristine (a) OCF and (b) BC.

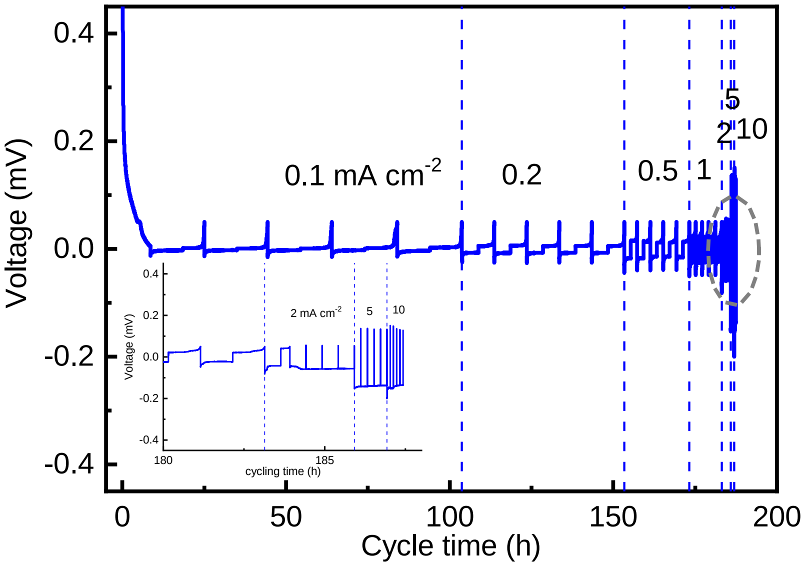

Figure S3. Rate performance of BC at various current densities.

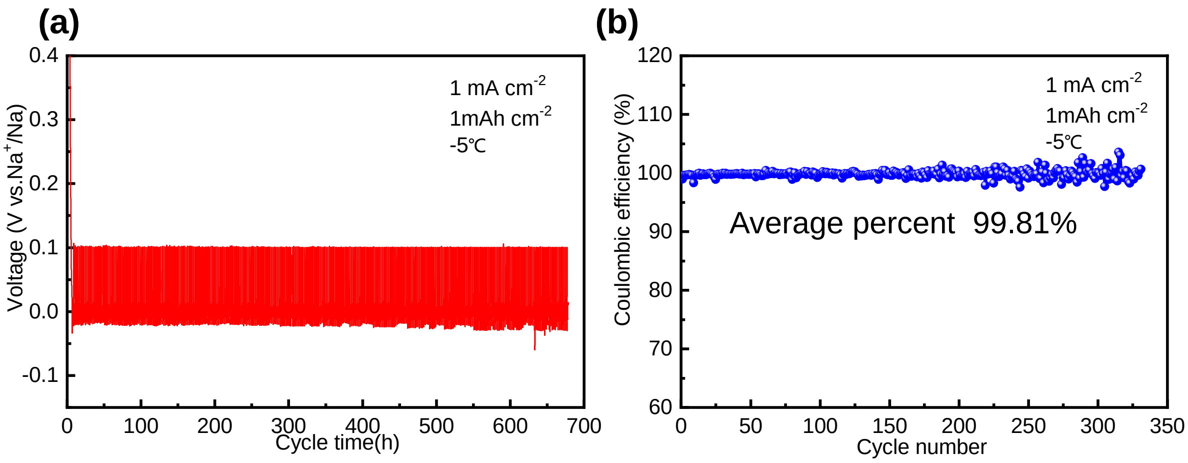

Figure S4. (a) Cycling performances of OCF and (b) coulombic efficiency of OCF at a current density of 1 mA cm^{-2} with a capacity of 1 mAh cm^{-2} at -5°C.

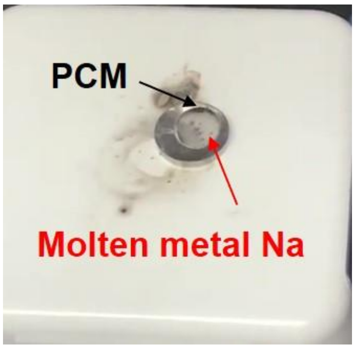

Figure S5. The surface wettability experiment on OCF surface.

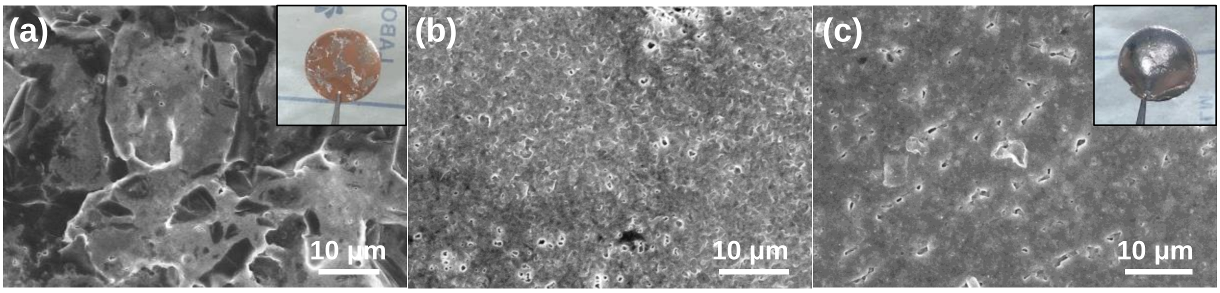

Figure S6. Top-view SEM images of Na metal on the Cu electrode with different plating capacity (a) 0.1 mAh cm^{-2}, (b) 0.5 mAh cm^{-2}, (c) 2.0 mAh cm^{-2} at the current density is 1.0 mA cm^{-2}.

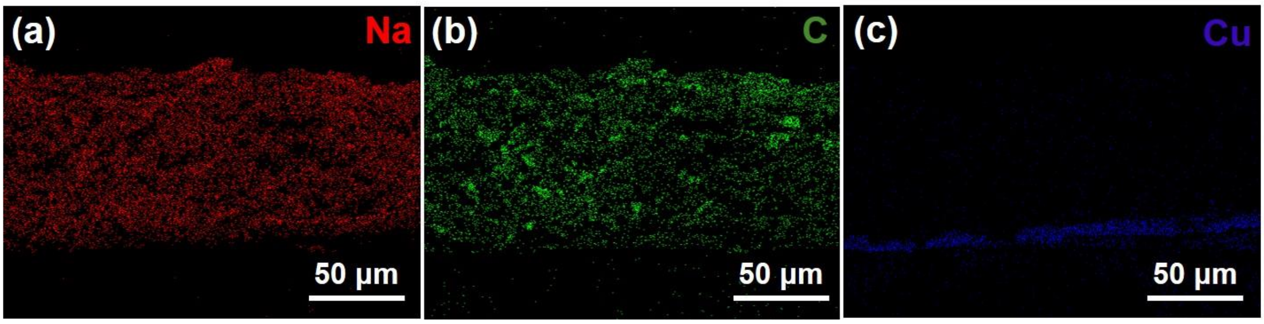

Figure S7. EDS element distribution maps of the elements (a) Na, (b) C and (c) Cu of the OCF electrode when the plating capacity is 2 mAh cm^{-2}

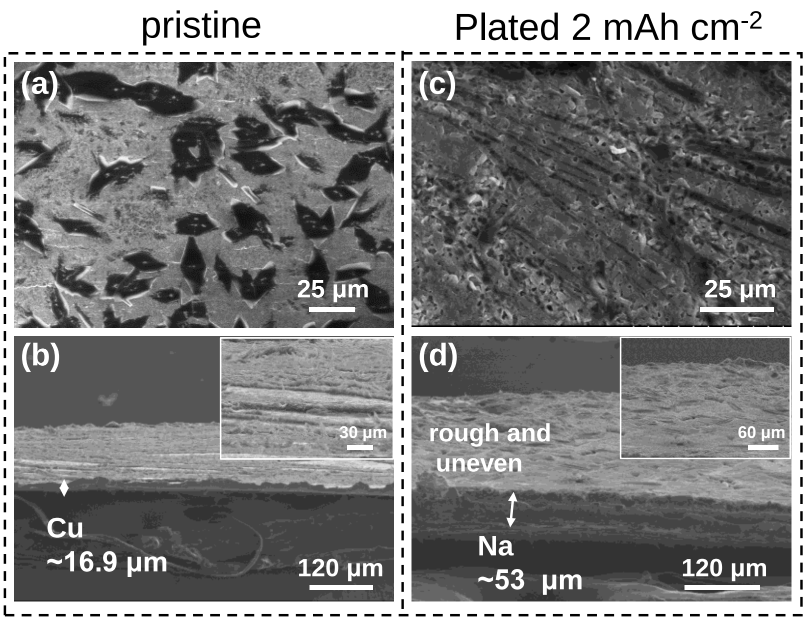

Figure S8. SEM characterization on the morphological evolution of Cu electrodes of (a) (c) pristine, (b) (d) after Na plating for 2 mAh cm^{-2}.

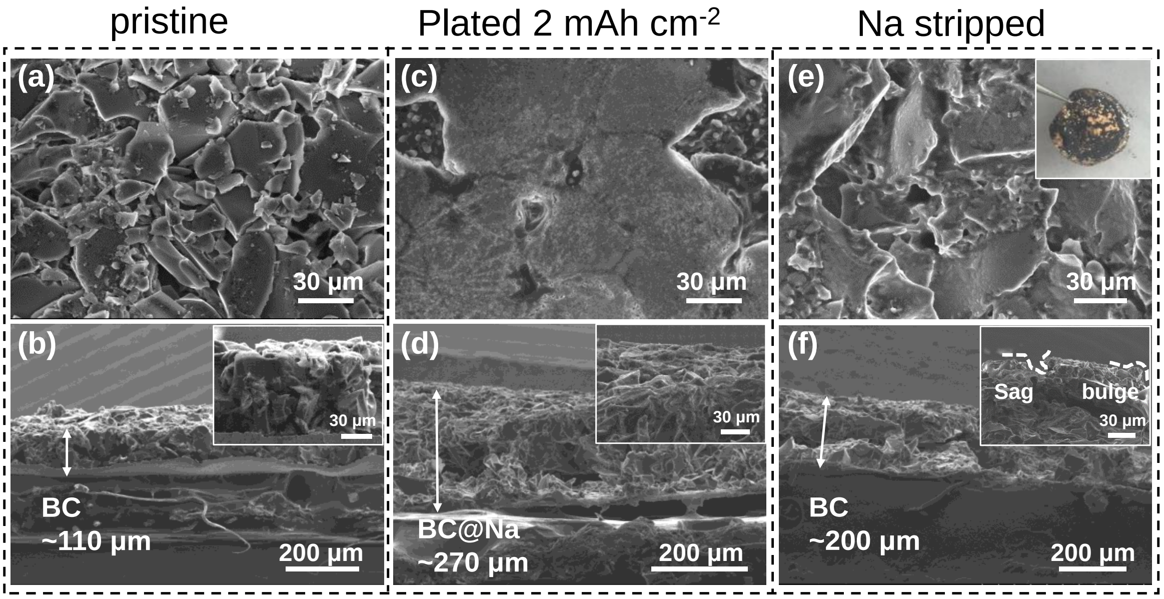

Figure S9. SEM characterization and optical pictures on the morphological evolution of BC electrodes of (a) (b) pristine, (c) (d) after Na plating for 2 mAh cm^{-2}, (e) (f) after Na stripping for 2 mAh cm^{-2}.

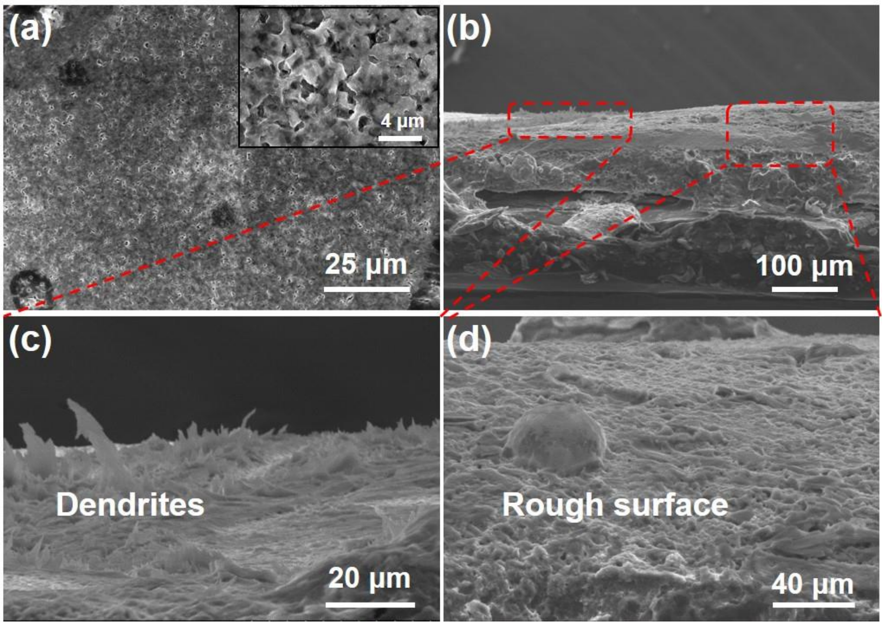

Figure S10. SEM images of the Cu electrode after 50 cycles with a current density of 5 mA cm^{-2} and a plating capacity of 5 mAh cm^{-2}(a) (b) (c) (d).

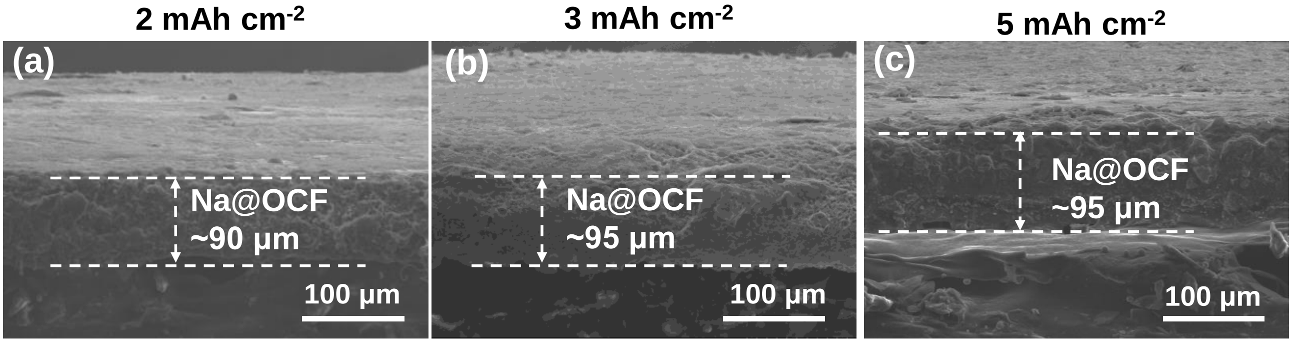

Figure S11. Cross-section view SEM images of the OCF electrode with a current density of 1 mA cm^{-2} and a plating capacity of (a) 2 mAh cm^{-2}, (b) 3 mAh cm^{-2}, (c) 5 mAh cm^{-2}.

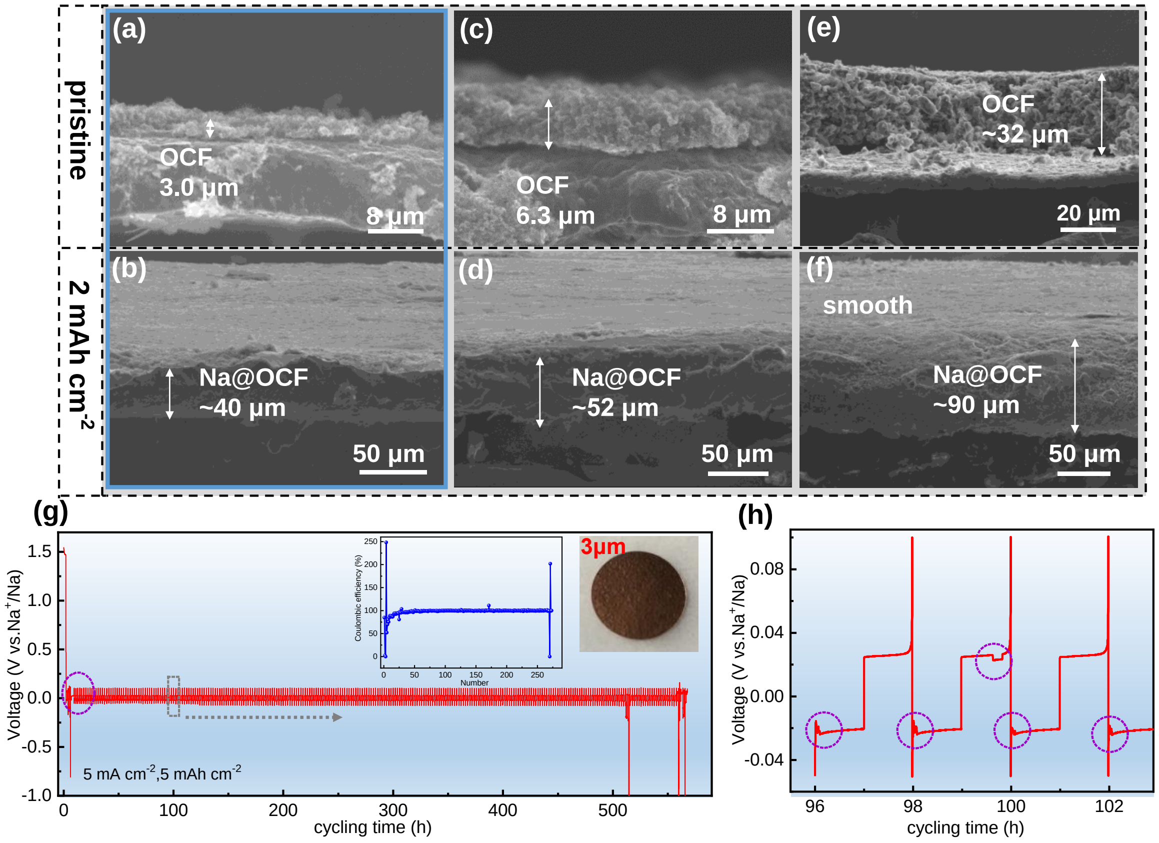

Figure S12. SEM cross-sectional view of different thicknesses of OCF electrodes (a) (c) (e) and when the deposition capacity was 2 mAh cm^{-1}under different electrode thicknesses (b) (d) (f), (g) cycling performances of OCF with thickness of 3 μm at a current density of 5 mA cm^{-2} with a capacity of 5 mAh cm^{-2} , (h) Details of voltage curve profiles when cycling time was 96-102 h.

We used ultrasonic spraying to precisely control OCF electrodes of different thicknesses, and compared the important role of OCF electrodes in the metal sodium plating/stripping process.[1] When the current density was 5.0 mA cm^{-2} and the plating capacity was 5.0 mAh cm^{-2}, we found that the voltage curve of OCF electrode (3 μm) showed obvious fluctuation and a short circuit occurred after ~270 cycles, which was completely different from the ultra-long cycle stability of OCF electrode with thickness of ~30 μm under large current and deposition capacity (Figure 5f). It can be understand that the nucleation size is small at high current density, metal Na is filled in OCF netlike pore structure, fewer OCF layers can only accommodate a certain

amount of metal Na. Once the deposition capacity continues to increase, multiple plating/stripping processes will inevitably lead to the incompleteness of the electrode, which is not conducive to continuing to regulate the growth of sodium metal and form sodium dendrites. Besides, we used ex-situ SEM to observe the thickness changes of different OCF layers after metal Na plating, and find that the thickness of metal Na layers of OCF electrodes with different thicknesses varies greatly at the same plating capacity, which indicated that the thickness of Na layer was related to the thickness of OCF layer. In summary, we believe that the OCF electrode has the function of accommodating the deposited metal Na, which relieves the infinite volume change during the cycling of metal Na anodes.

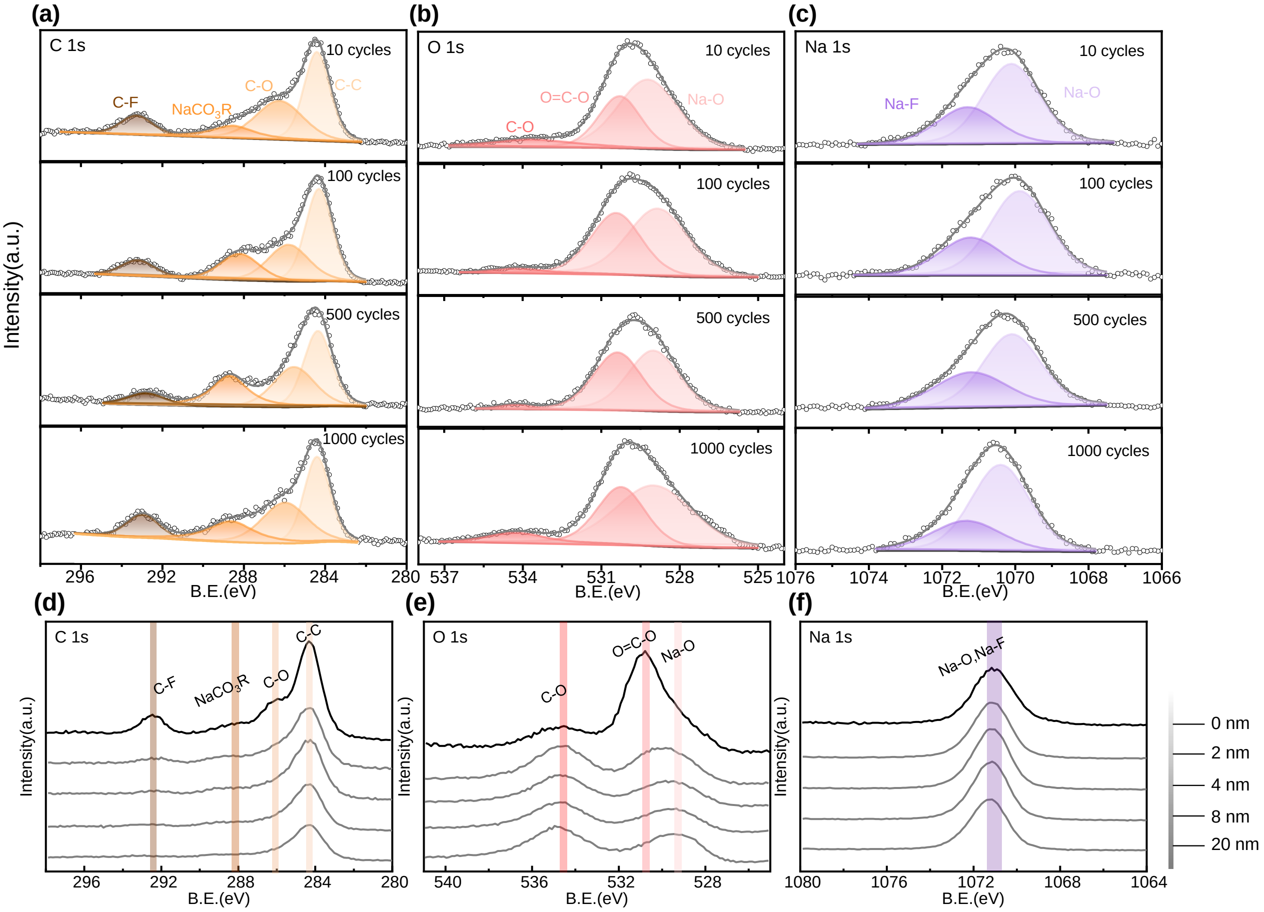

Figure S13. Ex-situ XPS of OCF electrode (a) C 1s spectra; (b) O 1s spectra; (c) Na 1s spectra after various galvanostatic plating/stripping cycles, (d) C 1s spectra; (e) O 1s spectra; (f) Na 1s spectra after 10 cycles at 1.0 mA cm^{-2}, 2.0 mAh cm^{-2} with depth profiles.

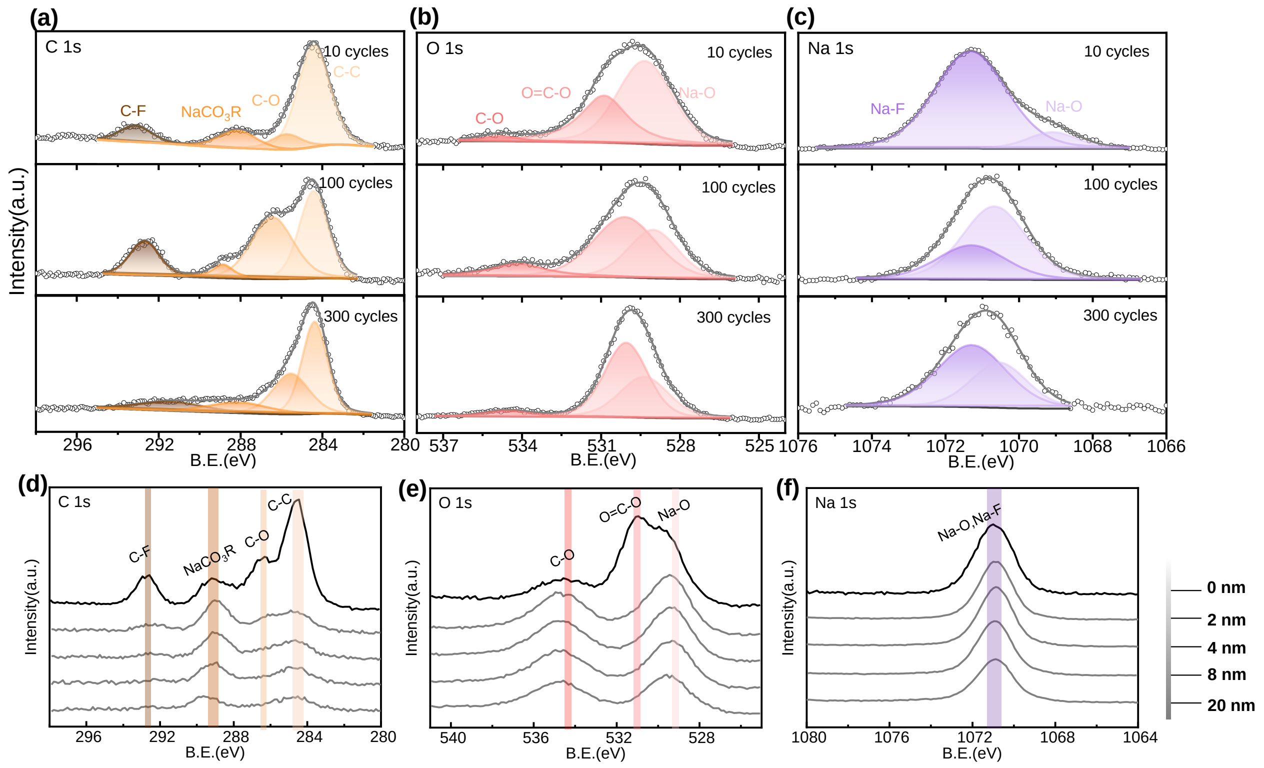

Figure S14. Ex-situ XPS of Cu electrode (a) C 1s spectra; (b) O 1s spectra; (c) Na 1s spectra after various galvanostatic plating/stripping cycles, (d) C 1s spectra; (e) O 1s spectra; (f) Na 1s spectra after 10 cycles at 1.0 mA cm^{-2}, 2.0 mAh cm^{-2} with depth profiles.

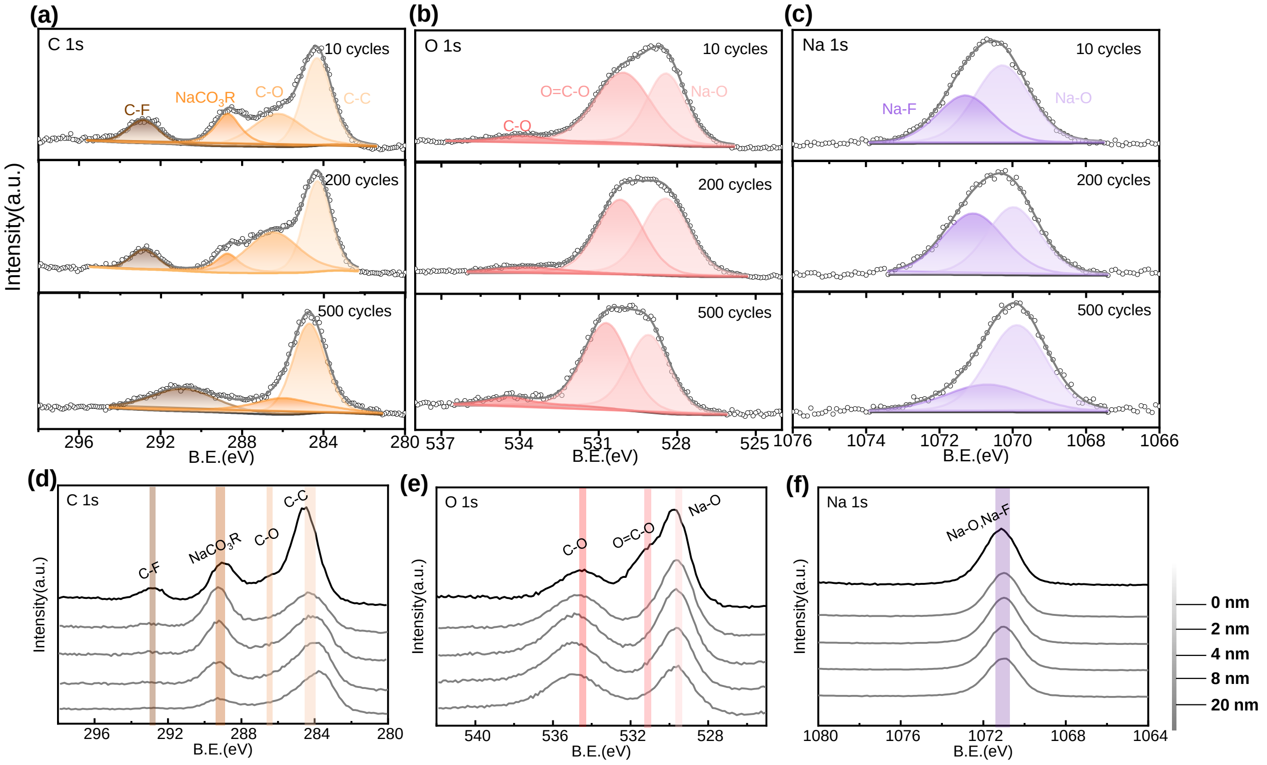

Figure S15. Ex-situ XPS of BC electrode (a) C 1s spectra; (b) O 1s spectra; (c) Na 1s spectra after various galvanostatic plating/stripping cycles, (d) C 1s spectra; (e) O 1s spectra; (f) Na 1s spectra after 10 cycles at 1.0 mA cm^{-2}, 2.0 mAh cm^{-2} with depth profiles.

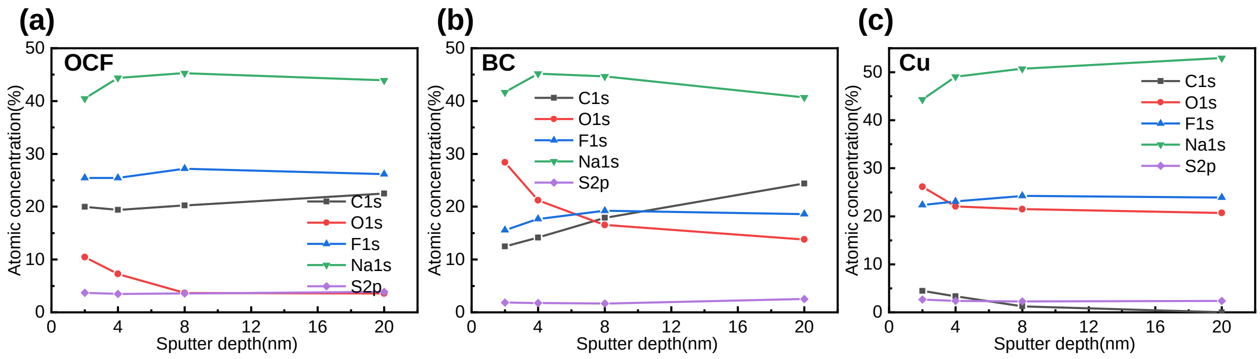

Figure S16. The elemental ratios at different depths of the SEI on (a) OCF, (b) BC and (c) Cu electrode.

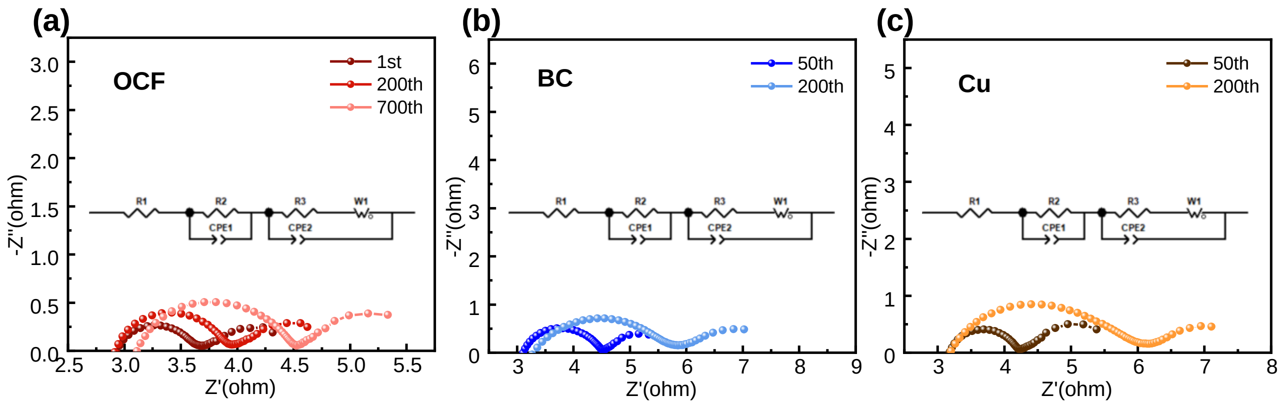

Figure S17. Impedance spectra of (a) OCF after sodium plating at a current density of 1 mA cm^{-2} with a capacity of 1 mAh cm^{-2} at the 1st, 200th and 700th cycle state; (b) BC after sodium plating at a current density of 1 mA cm^{-2} with a capacity of 1 mAh cm^{-2} at the 50th, 200th cycle state; (c) Cu foil after sodium plating at a current density of 1 mA cm^{-2} with a capacity of 1 mAh cm^{-2} at the 50th, 200th cycle state.

Table S1. The impedance data of the OCF, BC and Cu electrodes.

R (Ω)	R_s [a]	R_{SEI} [b]	R_{ct} [c]
OCF-1st	2.96	0.67	1.04
OCF-200th	2.96	0.95	1.11
OCF-700th	3.13	1.37	1.28
BC-50th	3.16	1.36	1.20
BC-200th	3.29	2.48	1.92
Cu-50th	3.23	0.96	1.50
Cu-200th	3.13	2.87	2.11

[a] Solution resistance between the lithium anode and the cathode; [b]The resistance of electrode solid electrolyte interface (SEI); [c] Charge-transfer resistance of the electrochemical reaction.

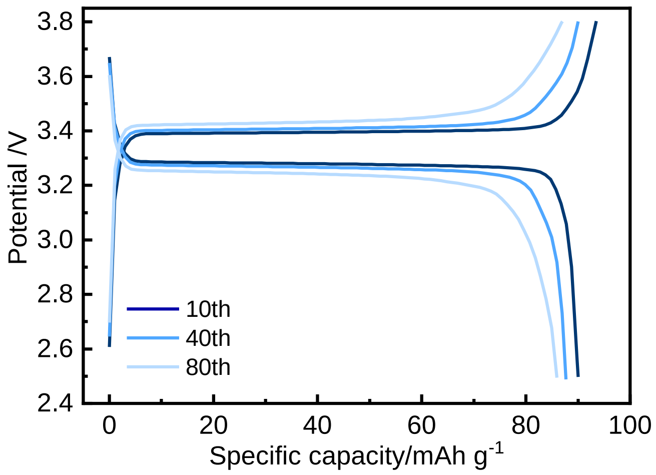

Figure S18. The voltage profiles of BMC@Na||Na_3V_2 $(PO_4)_3$ full cells.

Table S2: A summary of coulombic efficiency between previous and this work

Materials	Current density (mA cm^{-2})	Areal capacity (mA h cm^{-2})	Cycle numbers	Coulombic efficiency (%)	Electrolyte	References
Cu foil	0.5	1	300	99.9	1 M $NaPF_6$ diglyme	[2]
carbon/Al	0.5	0.25	1000	99.8	1 M $NaPF_6$ diglyme	[3]
Cu	1	1	280	98.3	1 M NaOTf diglyme +0.01 M KTFSI	[4]
	0.5	1	145	98.5	1 M NaOTf diglyme+ 0.01 M NaTFSI	
	0.5	1	100	93.8	1 M NaOTf diglyme	
heteroatom-doped (N,S) hollow carbon fibers	1	1	600	99.52	1 M CF_3NaO_3S in diglyme	[5]
N and O co-doped graphitized carbon fibers	2	8	120	99.8	1 M $NaPF_6$ diglyme	[6]

Sodiophilic M–Na alloys<br>Cu@Sn-NPs<br>Cu@Sb-MPs<br>Cu@Au-film	2<br>2<br>2	2<br>2<br>1	600<br>600<br>2000	99.8<br>99.9<br>99.9	0.01 M NaTFSI<br>+<br>1 M CF_3NaO_3S<br>in diglyme	[7]
oxygen-functionalized carbon nanotube network	1<br>5	1<br>10	3000<br>680	99.7<br>99.5	1 M CF_3NaO_3S<br>in diglyme	[8]
Sn^{2+} pillared Ti_3C_2 MXene	4<br>5<br>10	4<br>5<br>3	500<br>200<br>100	98.9<br>98.8<br>98.5	1 M $NaPF_6$<br>diglyme	[9]
This Work	10<br>5	1<br>5	2500<br>2000	99.83<br>99.90	0.01 M NaTFSI<br>+<br>1 M CF_3NaO_3S<br>in diglyme	

References

[1] a) A. D. Willey, J. M. Holt, B. A. Larsen, J. L. Blackburn, S. Liddiard, J. Abbott, M. Coffin, R. R. Vanfleet, R. C. Davis, *Journal of Vacuum Science & Technology B, Nanotechnology and Microelectronics: Materials, Processing, Measurement, and Phenomena* **2014**, 32; b) X. Wen, K. Xiang, Y. Zhu, L. Xiao, H. Liao, W. Chen, X. Chen, H. Chen, *J. Alloys Compd.* **2020**, 815, 152350.

[2] Z. W. Seh, J. Sun, Y. Sun, Y. Cui, *ACS Cent. Sci.* **2015**, 1, 449.

[3] A. P. Cohn, N. Muralidharan, R. Carter, K. Share, C. L. Pint, *Nano Lett.* **2017**, 17, 1296.

[4] Q. Shi, Y. Zhong, M. Wu, H. Wang, H. Wang, *Angew. Chem. Int. Ed.* **2018**, 57, 9069.

[5] X. Zheng, P. Li, Z. Cao, W. Luo, F. Sun, Z. Wang, B. Ding, G. Wang, Y. Huang, *Small* **2019**, 15, 1902688.

[6] Z. Zheng, X. Zeng, H. Ye, F. Cao, Z. Wang, *ACS Appl Mater Interfaces* **2018**, 10, 30417.

[7] S. Tang, Y. Y. Zhang, X. G. Zhang, J. T. Li, X. Y. Wang, J. W. Yan, D. Y. Wu, M. S. Zheng, Q. F. Dong, B. W. Mao, *Adv. Mater.* **2019**, 31, 1807495.

[8] L. Ye, M. Liao, T. Zhao, H. Sun, Y. Zhao, X. Sun, B. Wang, H. Peng, *Angew. Chem. Int. Ed.* **2019**, 131, 17210.

[9] J. Luo, C. Wang, H. Wang, X. Hu, E. Matios, X. Lu, W. Zhang, X. Tao, W. Li, *Adv. Funct. Mater.* **2019**, 29, 1805946.
